# The effect of climate variables on the incidence of Crimean Congo Hemorrhagic Fever (CCHF) in Zahedan, Iran

**DOI:** 10.1186/s12889-020-09989-4

**Published:** 2020-12-09

**Authors:** Sairan Nili, Narges Khanjani, Yunes Jahani, Bahram Bakhtiari

**Affiliations:** 1 Neurology Research Center, Kerman University of Medical Sciences, Kerman, Iran; 2 Environmental Health Engineering Research Center, Kerman University of Medical Sciences, Kerman, Iran; 3 Modelling in Health Research Center, Institute for Future Studies in Health, Kerman University of Medical Sciences, Kerman, Iran; 4 Water Engineering Department, College of Agriculture, Shahid Bahonar University, Kerman, Iran

**Keywords:** Iran, Forecasting, Time-series analysis, SARIMA, Generalized additive model, Hemorrhagic fever, Crimean-Congo

## Abstract

**Background:**

The Crimean-Congo Hemorrhagic fever (CCHF) is endemic in Iran and has a high fatality rate. The aim of this study was to investigate the association between CCHF incidence and meteorological variables in Zahedan district, which has a high incidence of this disease.

**Methods:**

Data about meteorological variables and CCHF incidence was inquired from 2010 to 2017 for Zahedan district. The analysis was performed using univariate and multivariate Seasonal Autoregressive Integrated Moving Average (SARIMA) models and Generalized Additive Models (GAM) using R software. AIC, BIC and residual tests were used to test the goodness of fit of SARIMA models, and R^2^ was used to select the best model in GAM/GAMM.

**Results:**

During the years under study, 190 confirmed cases of CCHF were identified in Zahedan district. The fatality rate of the disease was 8.42%. The disease trend followed a seasonal pattern. The results of multivariate SARIMA showed the (0,1,1) (0,1,1)_12_ model with maximum monthly temperature lagged 5 months, forecasted the disease better than other models. In the GAM, monthly average temperature lagged 5 months, and the monthly minimum of relative humidity and total monthly rainfall without lag, had a nonlinear relation with the incidence of CCHF.

**Conclusions:**

Meteorological variables can affect CCHF occurrence.

## Background

Crimean-Congo hemorrhagic fever (CCHF) is a viral disease with a worldwide distribution [1]. It is transmitted by ticks or through contact with infected animal tissue. The most important route of CCHF transmission in Iran is contact with the blood or tissues of infected domestic animals. The length of incubation period depends on the viral load, the route of transmission, and the source of infection. After tick bites, the incubation period is 1–5 days and a maximum of 9 days, but following contact with infected blood or tissue, the incubation period is 5–7 days and a maximum of 13 days. The minimum viral load for disease transmission is 1 to 10 organisms [2]. Attention to this disease has increased, because of its severe and numerous outbreaks in recent years, its spread as an epidemic, and its high fatality rate which is 10–40%. CCHF is now endemic in Africa, Central Asia, Russia, China and the Balkans up to 48 degrees latitude [3]; and its incidence is rising rapidly in the Eastern Mediterranean region [4].

Iran is one of the endemic regions and has a high incidence of CCHF [5]; and needs to be prepared for disease control. Therefore, it is important to predict where and when the next cases of disease will occur [6]. Rising global temperatures have increased the duration of hot and dry weather, and this can result in the spread and increase of the population of vector ticks that carry the CCHF virus [7, 8]. Also, following temperature changes, migratory birds facilitate the spread of tick species, especially hyalomma [9]. Ticks prefer warm and dry climates. Therefore, increasing temperature and decreasing rainfall provide a good environment for ticks to grow and reproduce [10, 11]. Changes in agricultural land, and landscape alteration following climate change may also influence the survival and reproduction of ticks, and may trigger disease outbreaks [12].

As climate change has affected many regions around the world, it is necessary to study its impact on vector-borne diseases. Studies have shown that the Sistan and Baluchistan province is one of the most prominent endemic areas for CCHF in Iran [13–15]. The residents of Zahedan are largely from Baloch ethnicity. This province has an arid climate based on the De Martonne classification.

Appropriate management actions can be taken to reduce the burden of CCHF in the future, in Iran, if accurate predictions are made. The aim of this study was to investigate the association between CCHF incidence and meteorological variables in Zahedan district, which has a high incidence of this disease.

## Methods

### Research design

This was an ecological study based on available CCHF incidence data and meteorological variables.

### Study population and area

Zahedan is the capital of Sistan and Baluchistan province. It is located in southeastern Iran, near the Afghanistan and Pakistan border. Geographically, it is located in 60° 52′ 00˝ E and 29° 29′ 45˝ N [16]; and has the highest incidence of CCHF in Iran [5]. The population of Zahedan city according to the 2016 national census was 587,730 persons.

### Outcome data

In Iran, cases of CCHF have to be reported immediately, by telephone to district and provincial health centers, and the Iranian Ministry of Health and Medical Education’s Center for Communicable Disease Control. Samples are routinely taken from potentially infected individuals and are sent to the Arboviruses and Viral Hemorrhagic Fevers Laboratory at the Pasteur Institute laboratory of Iran. A potentially infected individual is a person showing acute symptoms of the disease with a history of traveling to rural areas, contact with livestock or tick bites. For ease of diagnosis, the criteria suggested by Swanepoel and Harvey 1987, which is based on history of contact, clinical signs and laboratory findings, is used. If the total score obtained from the CCHF diagnostic criteria tables is 12 or more, the patient is considered as a probable case [17]. The confirmed cases are identified based on detection of specific IgM antibodies, a 4-fold rise in the titer of IgG antibodies, or the detection of viral antigens by reverse transcriptase PCR (RT-PCR) [18]. The data recorded by the city health center is later reviewed by the provincial health center and the Communicable Disease Management Center at the Ministry of Health, and is matched with the statistics sent by the Pasteur Institute. Therefore, the possibility of overcounting or undercounting is very low. The proposal of this study was reviewed at Kerman University of Medical Sciences and ethics approval was granted (Ethic Code: IR.KMU.REC.1397.232). After approval, an official letter was sent to the Center for Communicable Diseases Management, at the Ministry of Health and Medical Education, Tehran, Iran; for requesting information about CCHF incidence. This information was handed to the first author, as aggregated data, without any personal identity. As Researchers did not know the identity of the patients, their consent was not needed.

The dependent outcome variable in this study was the confirmed cases of CCHF, in Zahedan in 2010–2017. The number of cases diagnosed monthly was used in the statistical models.

In case the data of some variables was missing, the researchers would contact the Zahedan deputy of health and information was completed based on patient records. The recorded time of disease onset, which has been used in this study, was the time that the first symptoms appeared, not the time the diagnosis was made.

### Meteorological data

Eight potentially related synoptic meteorological variables were inquired from the Zahedan city meteorological station. These variables included average, minimum and maximum of daily temperature (°C), mean, minimum and maximum of relative humidity (%), daily and monthly cumulative rainfall (in millimeters) and sunshine (sum of hours per day and month). In case data was not available for some days, the monthly average was calculated based on data from the available days. Meteorological variables were inquired from national meteorological organization from 2010 until 2017.

### Modeling approach and evaluation

SARIMA time series models were used, and these steps were followed; 1) plotting the data, 2) transforming data if necessary 3) identifying the preliminary autoregressive, differencing and moving average values (p, d, q) 4) parameter estimation, 5) diagnostics, and 6) model selection.

Initially the time series graph of CCHF cases was plotted. The Dicky-Fuller test was used to assess non-stationary of the mean. Then non-seasonal (d) and seasonal differences (D) were used to convert non-stationary to stationary data. Box-Cox transformations were used to stabilize the variance. The Auto Correlation Function (ACF) and Partial Auto Correlation Function (PACF) graphs were used to identify the trend, seasonality and stationery. By checking these graphs, the Moving Average (MA) and Auto Regressive (AR) parameters were estimated and the P, p and Q, q were identified, respectively.

In the diagnosis step, the residuals were tested for independence and normality by building the histogram of residuals or the Q-Q plot. In the final step, the best model was selected according to the lowest Akaike information criterion (AIC), AIC corrected for sample size (AICc) and Bayesian information criterion (BIC) [19]. BIC is a more conservative test than AIC. AIC or BIC can be preferable, depending on the relative importance one assigns to sensitivity versus specificity. In larger samples sizes AIC considers sensitivity more than specificity. Also in general, AIC prefers models with more parameters and BIC with less [20]. However, in this study, the AIC and BIC results were consistent in all models and the best fitted model was determined according to both the lowest AIC and BIC.

Multivariate time series analysis was used to investigate the dynamic relation between climate variables and CCHF incidence [19]. In this model the cross-correlation functions (CCF) were calculated, and effective variables and their best lags were identified. The pre-whitening procedure was used to remove the auto-correlation and seasonal trend. Due to the existence of a collinearity between some climate variables, the Variance Inflation Factor (VIF) was calculated and variables that had an VIF < 10 were entered with different lags. VIF is an index that measures how much the variance (the square of the estimate’s standard deviation) of an estimated regression coefficient is increased because of collinearity [21]. VIF values greater than 10 reveal multicollinearity issues [22].

We used “tseries” package version 0.10–47 designed by Trapletti [23] in R software (R version 3.5.3) to do the statistical analysis.

We also used Quasi-Poisson Generalized Additive Models (GAM) which are semi-parametric extensions of generalized linear models with predictors including a sum of smooth functions of covariates. In general, the model has a structure like

$$ g\left(\mu i\right)=\upbeta 0+f1(X1i)+f2(X2i)+f3(X3i)+\upvarepsilon \mathrm{i}\dots $$

were g (μi) is a link function, fi (Xi) are smooth functions for covariates Xi, and ε is random effect. Generalized additive models (GAMs) provide an extension to standard linear models by allowing non-linear functions for each of the variables, while maintaining additivity. Just like linear models, GAMs additivity can be applied with both quantitative and qualitative responses. In order to allow for non-linear relations between each feature and the response, each linear component βj (xij) is replaced with a (smooth) nonlinear function fj (xij) [24]. GAMs automatically identify and estimate the optimal degree of nonlinearity of the model directly from the data [25]. In this model, we also computed CCFs between the Y and Xi series, and VIFs for selecting variables and their lag(s) in the model.

Eventually, because the observations had high dispersion and autocorrelation, we used the Generalized Additive Mixed Model (GAMM) instead of GAM. GAMM models allow flexible dependence of a response variable on independent variables, by using nonparametric regression; and also includes correlations between observations in the model by using random effects [24]. R^2^ was used to select the best model.

The “mgcv” package version 1.8–7, designed by Wood [25] available in R statistical software (version 3.5.3) was used for analysis.

## Results

Overall, 190 cases of CCHF had been reported between 2010 and 2017 from Zahedan district. 88% were male, and their mean age was 31.9(±13.59). The majority of cases (88%) had a history of contact with livestock; 65% of cases were ranchers, abattoir workers or butchers; and 90% lived in the city. 9% of the cases were Afghan and only one case was from Pakistan, and the rest were Iranian. The case fatality rate (CFR) of CCHF in this study was 8.42%. CFR was not significantly different in gender (*p* = 0.42) or residence (urban, rural) subgroups (*p* = 0.40).

**Fig. 1:**
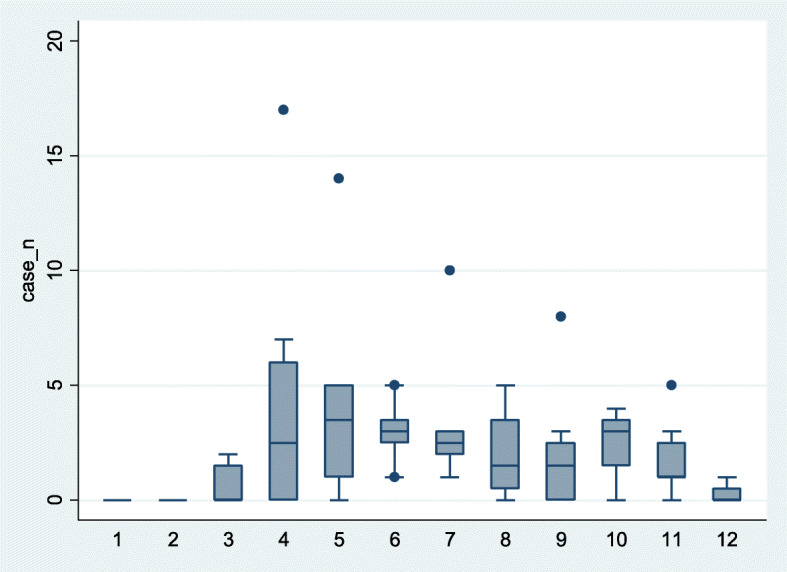
Box plot of monthly CCHF incidence

The monthly distribution of CCHF cases in Zahedan (Fig. 1), shows that incidence had a seasonal pattern; and the incidence of disease differed significantly in various months (Kruskal-Wallis = 42.19, df = 11, *p* < 0.001).

**Fig. 2:**
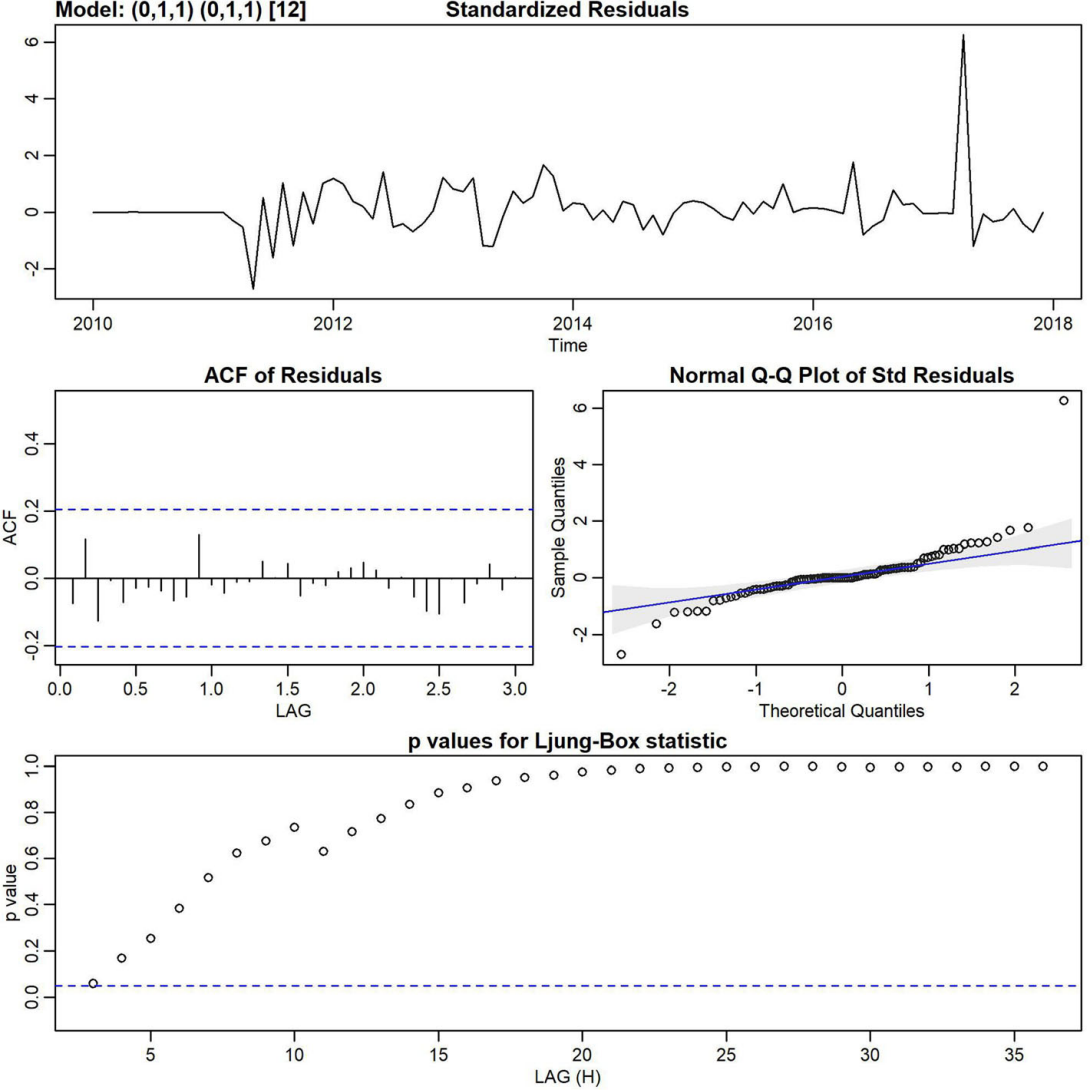
Diagnostics of the residuals from SARIMA (0,1,1) (0,1,1)_12_

**Fig. 3:**
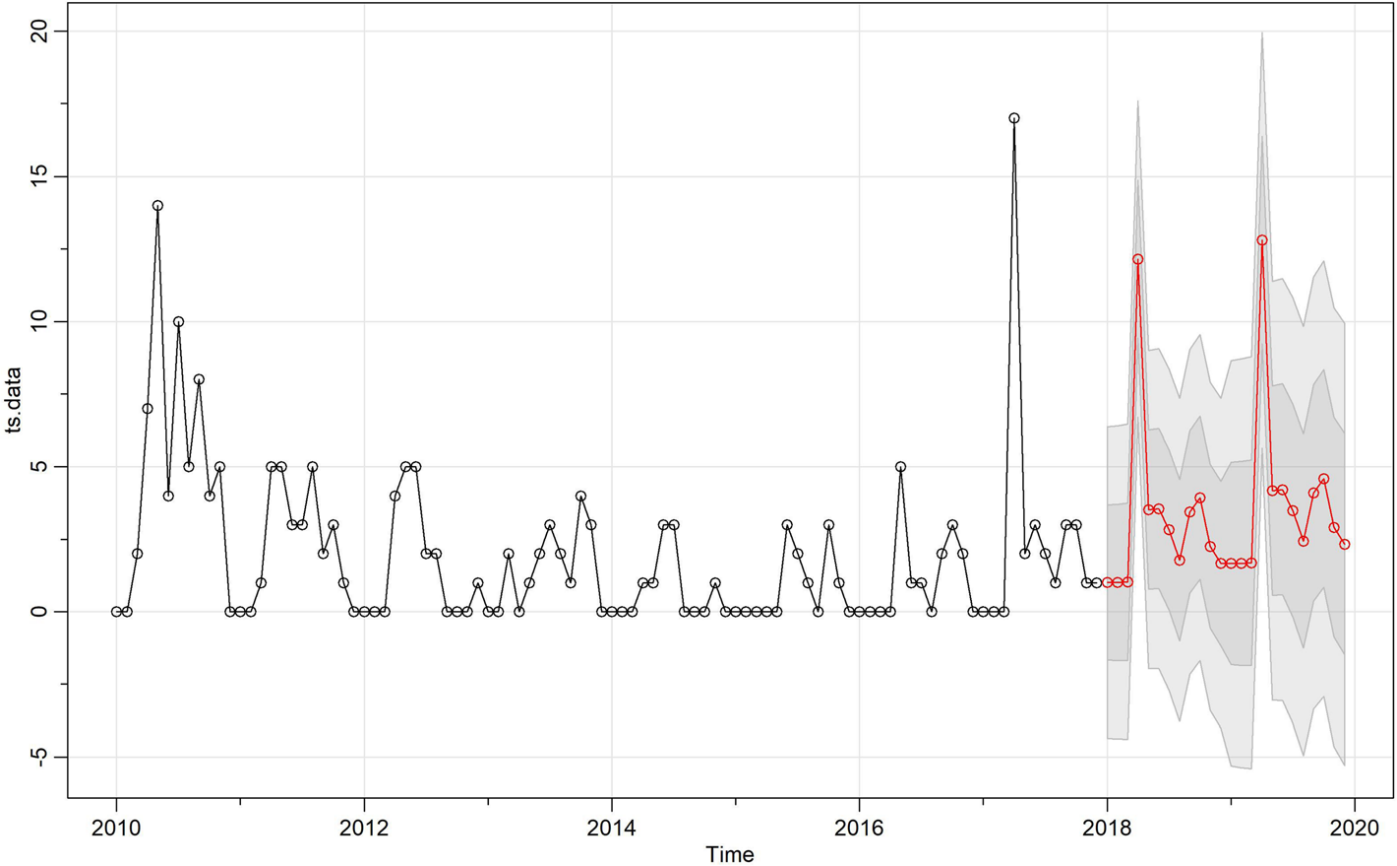
The best-fitting model according to AIC/BIC and predictions for 2018–2020

Inspection of the time plot of standardized residuals (Fig. 2) showed no obvious pattern. The normal Q-Q plot of the residuals showed that the assumption of normality was reasonable. As shown in Table 1, different models were built and the SARIMA (0,1,1) (0,1,1)_12_ univariable model had the lowest AIC and BIC (AIC = 4.34, BIC = 4.42). Based on the Ljung-Box test, residuals did not have significant autocorrelations (Fig. 2). Figure 3 illustrates the best fitting model.

**Table 1:** Test results comparing the performance of the constructed models

Model	AIC	BIC
MA(1)	4.89	4.97
MA(2)	4.84	4.94
SARIMA(0,1,1)(0,1,1)	4.34	4.42
SARIMA(1,0,1)(0,1,1)	4.36	4.49
SARIMA(1,0,2)(0,1,1)	4.38	4.53
SARIMA(1,2,2)(0,1,1)	4.52	4.68
multivariate SARIMA(0,1,1)(0,1,1)	4.34	4.71
multivariate SARIMA(1,0,1)(0,1,1)	4.34	4.74
multivariate SARIMA(1,0,2)(0,1,1)	4.45	4.78
multivariate SARIMA(1,2,2)(0,1,1)	4.59	4.93

### Meteorological predictors

As seen in Table 2, the zero lag climate variables were examined in the null model, with the lowest AIC and BIC, but none of the variables were related to the incidence of CCHF. In the multivariate model, according to the Variance Inflation Factor (VIF ≤10) and the CCF, appropriate variables and their lags were chosen, and the SARIMA (0,1,1) (0,1,1)_12_ with maximum temperature lagged 5 months, had the lowest AIC and BIC and was the best model to predict CCHF incidence (AIC = 4.10, AIC_C_ = 4.10, BIC = 4.20) (Table 3).

**Table 2:** Comparison of candidate SARIMA models for number of CCHF cases in Zahedan district, Iran

Model	Variables		Lag	Estimate	SE	t	***P***-value
[A]	Monthly patients	Constant		1.96	0.40	4.81	< 0.001
		MA	Lag 1	0.26	0.09	2.75	0.007
			Lag 2	0.26	0.09	2.77	0.006
[B]		MA	Lag 1	−0.89	0.04	−18.57	< 0.001
		MA, seasonal	Lag 1	−0.34	0.17	−1.95	0.05
[C]		MA	Lag 1	−0.87	0.05	− 14.82	< 0.001
		Seasonal difference		1			
		MA, seasonal	Lag 1	−0.37	0.19	−1.95	0.054
	Maximum temperature			0.023	0.14	0.16	0.86
	Minimum temperature			0.26	0.24	1.09	0.27
	Mean temperature			−0.24	0.18	−1.33	0.18
	Rain fall			0.02	0.04	0.57	0.56
	Maximum Humidity			−0.01	0.02	− 0.76	0.44
	Minimum Humidity			−0.19	0.13	−1.46	0.14
	Mean Humidity			0.06	0.06	0.92	0.35
	Sunshine			0.02	0.01	−1.33	0.18

[A]: MA (2), [B]: univariate SARIMA (0,1,1) (0,1,1), [C]: multivariate SARIMA (0,1,1) (0,1,1), SARIMA: seasonal auto-regressive integrated moving average

A) AIC: 4.84, AICc: 4.84, BIC: 4.94, log likelihood = − 228.45

B) AIC: 4.34, AICc: 4.34, BIC: 4.42, log likelihood = − 201.34

C) AIC: 4.43, AICc: 4.46, BIC: 4.71, log likelihood = − 197.43

**Table 3:** The best fitted SARIMA model for meteorological variables in Zahedan district, Iran

Model	Variables		Lag	Estimate	SE	T	*P*-value
	Monthly patients	MA	Lag 1	−0.89	0.048	−18.5	0.001
		Seasonal difference	1				
		MA, seasonal	Lag 1	−0.30	0.19	−1.54	0.12
	Maximum temperature		Lag 5	−0.26	0.11	−2.27	0.02

Log likelihood = −-188.86, AIC: 4.10, AICc: 4.10, BIC: 4.20

**Fig. 4:**
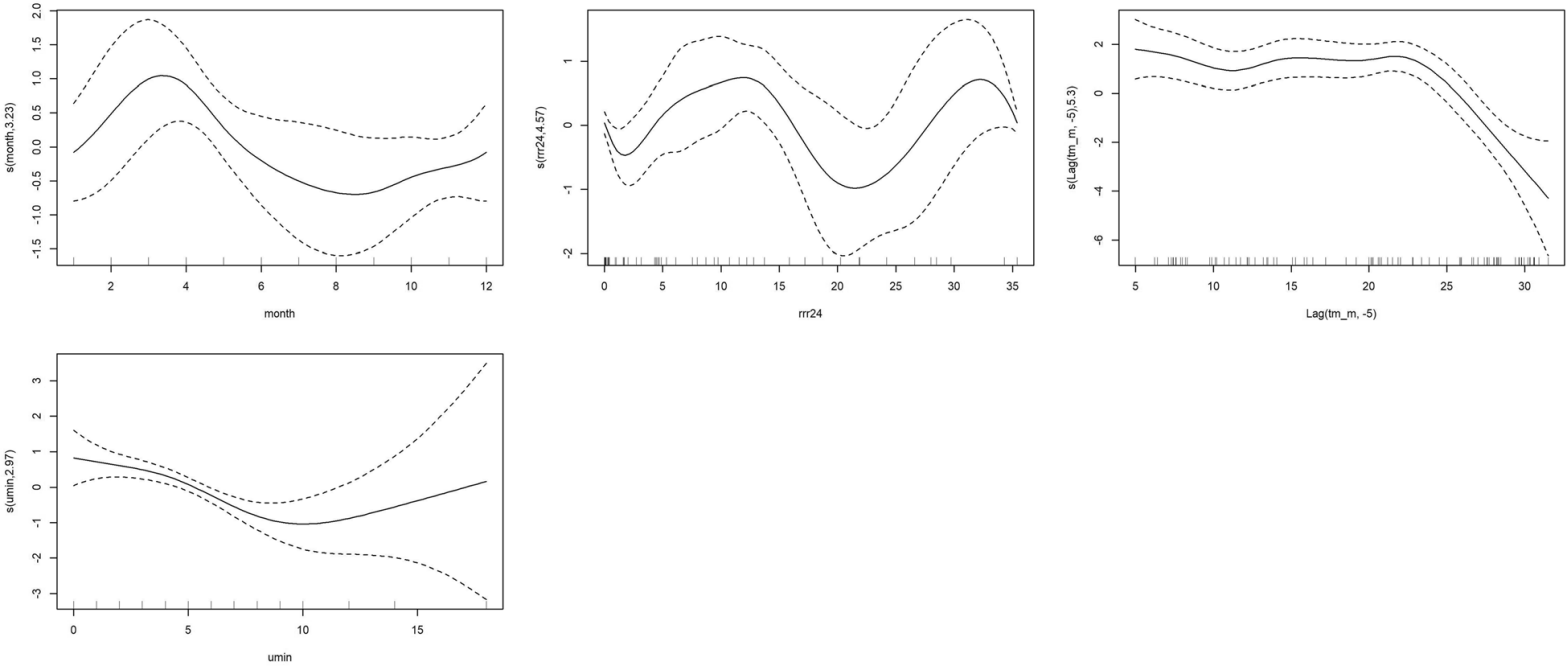
GAM-estimated relation between the number of CCHF cases and month (**a**), total monthly rainfall (**b**), monthly mean temperature (**c**), monthly minimum relative humidity (the lowest number recorded in the month) (**d**) The numbers on the vertical axis are the variable name and the effective degrees of freedom

In GAM, several models were run according to VIF and CCF. Results showed significant associations between CCHF incidence and monthly average temperature lagged 5 months, monthly minimum of relative humidity and total monthly rainfall without lag (Table 4). According to Fig. 4a, the month of the year showed a non-linear relation with CCHF incidence, with a positive effect from January to April, a negative effect from April to October and a positive effect from September until December. Figure 4b shows a wiggly association between total monthly rainfall and CCHF incidence (edf = 4.56), and a direct effect can be seen until 14 mm, then a negative effect between 14 and 20 mm and another direct effect until a peak at 33 mm. Mean monthly temperature lagged 5 months is shown in Fig. 4c and shows a nonlinear association that peaks at 22 °C and then decreases. An inverse effect for monthly minimum relative humidity was seen until 10%, and then a positive effect with wide uncertainty for over 10% (Fig. 4d). The best fitted GAM was:

$$ E(CCHF)=\beta 0+f1\left( month,i\right)+f2\left( average\ tempreture,5\right)+f3\left( minimum\ relative\ humidity\right)+f4(rainfall)\Big) $$

**Table 4:** Model estimates of effects of meteorological variables on CCHF incidence

Smooth terms	edf	F
S (month)	3.23	1.66***
s (Lag (average temperature, −5))	5.3	6.46***
s (minimum of relative humidity)	2.97	6.72***
s (rainfall)	4.56	1.8***
	Estimate	Std. Error
Intercept	−0.20	0.19
R-sq. (adj)	54%	

*** Significant at the 0.001 level

*edf* effective degrees of freedom of the smooth function term. Edf > 1 indicates nonlinear association

F value is an estimate of F-test

Other variables, including monthly maximum and minimum temperatures, hours of sunshine in month, and average and maximum relative humidity in month, were not significantly related and were removed from the model.

## Discussion

This study showed that the incidence of CCHF in Iran follows a seasonal pattern and its incidence is related to some meteorological variables. The case fatality rate (CFR) of CCHF in this study was 8.42%, which is lower than a previous study conducted on 2000 to 2015 data, which reported it was 14% in Iran [26], and a systematic review that estimated mortality was 33.5% in Asia [2]. The peak of this disease in Zahedan was between May to July. In other studies, the peak of CCHF incidence has been reported to be between April and September in Bulgaria [27], and mainly between April and August, in the south and southeast cities of Iran [14]. A national study conducted on 2000–2015 data in Iran showed that the increase in the incidence of CCHF started in the first month of summer and peaked in June [5]. Two annual peaks for CCHF were seen in Pakistan’s Baluchistan that has a common border with the east of Iran, which were in April and August [28], but another study from three public hospitals in Quetta in Baluchistan province of Pakistan, showed the highest number of hospitalizations for CCHF in Pakistan, occurred in June and July [29]. In Zabol and Zahedan city in Iran, most cases of CCHF occurred in the first 6 months of the year, which is the peak of tick activity [30]. In Turkey, which has a common border with western Iran, the peak of CCHF cases was seen around June and July and the number of patients was related to the previous year’s meteorological conditions which affected the vectors [12].

Studies have shown increasing populations of ticks and their related diseases in relation to climate change [31, 32] . Ticks usually live in warm temperatures [24]. In a study conducted in 2012 in Zahedan, Hyalomma and Haemaphysalis were the two main genera of hard ticks (Ixodidae) infected with CCHF virus [17]. Other studies reported that 46% of hyalomma spp. tick samples gathered in Iran were from desert climate, in Sistan and Baluchistan and Yazd provinces [33]. The Hyalomma tick is active in Iran from the beginning of spring and continues until the middle of summer [23].

Our result showed that the incidence of CCHF is related to some meteorological variables and this is consistent with previous studies done in the Eastern Mediterranean Region and Iran which will be discussed in the following paragraphs [12, 14, 27, 29]. However, a strength of this study over previous studies was that it used generalized additive models that were able to show non-linear relations, thresholds and ranges with different coefficients.

In this study, the multivariate SARIMA (0,1,1) (0,1,1)_12_ with monthly maximum temperature (the mean of the daily maximum temperatures) lagged 5-months, was the best prediction model; and also in GAM, the monthly mean temperature (the mean of the daily mean temperatures) with 5-months lag was related to the number of cases. In a study previously conducted in eastern Iran, a 1 °C increase in the maximum temperature after three months lag caused a 9% increase in the odds of CCHF occurrence [30]. Other studies showed that maximum temperature with 1 month lag had a positive correlation with CCHF cases in Zahedan, Iran [13] and there were significant direct associations between average temperature lagged one (r = 0.32) and two months (r = 0.36), and an inverse association between temperature lagged 5 months (r = − 0.43) with CCHF in Sistan-Baluchistan province, Iran [14]. According to the results of Ahmadkhani et al.’s study, the longevity of CCHF clusters in parts of Iran (lasting from early spring to late autumn) may be due to the relatively high average temperature in spring (more than 30 °C) and in autumn (almost 10 °C) [5]. According to the summary report of the First International Conference on Crimean-Congo hemorrhagic fever, most cases of the disease in Iran occur in the warm months, which is the peak of tick activity. In Turkey, outbreaks that occurred in the spring and summer of 2002, have been linked to a number of factors, including climate (evapotranspiration and temperature), geographic situation, and wildlife; and 60% of cases reported in June and July were probably due to global warming and earlier tick activity. In Bulgaria, most cases had occurred between April and November. In Russia and Central Europe, the spread of ticks in the warm and humid river valley played an important role in disease outbreaks; and in South Africa, cases occurred in the summer when adult hyalomma ticks are at their activity peak [34]. Another study from Iran showed there was a positive association between annual mean temperature and total tick abundance [33]; and in Bulgaria, the incidence of CCHF increased significantly as temperature increased [27]. The reason for the increased incidence of CCHF in warmer temperatures may be the increased number of ticks, people spending more time outdoors, and consequently the higher chance of getting bitten by ticks [33].

In this study, GAM results showed monthly minimum relative humidity (the lowest number recorded for humidity in the month) had a nonlinear relation with CCHF, but multivariate SARIMA did not show this relation. A study conducted previously in southeastern Iran, showed relative humidity with 1 and 6 month lags had a positive correlation with CCHF [13]; and another study from eastern Iran showed 1% increase in relative humidity, after 2 months caused a 4% increase in the odds of CCHF [30]. A study from eleven provinces in Iran showed there was generally a negative relation between precipitation and Hyalomma Ticks density [33].

In this current study, total monthly rainfall was related with CCHF and the peak of CCHF incidence was between 15 and 33 mm. In a previous study conducted in Iran, in univariate analysis, there was a significant relation between the incidence of CCHF and rainfall with a one-month (r = − 0.23) or five-month lag (r = 0.27), and in the best-fitted SARIMA regression model with a 5-month lag (β = 0.68; *p* = 0.001) [14]. Some studies have suggested an inverse relation between close rainfall and tick activity [17] and a direct relation after several months. Most species of the Ixodidae family are found in dry and semiarid environments, and the Hyalomma tick which is the main vector of CCHF, prefers arid zone vegetation, dry climates [10] and a place with numerous mammals for blood eating.

Environmental factors and human behavior are among the most important factors related to the continuation of the ticks’ life cycle [35]. According to ecological models ran by the World Health Organization Eastern Mediterranean Region (WHO EMR), increasing temperature and decreasing rainfall will expand the suitable habitat for hyalomma ticks and will subsequently increase CCHF [4]. Numerous studies have linked increase in the average annual temperature and decreased annual precipitation and relative humidity with increased CCHF occurrence [30, 36].

## Conclusion

Meteorological variables including monthly average temperature, monthly cumulative rainfall and minimum of relative humidity can affect CCHF occurrence in Zahedan, Iran.

## Data Availability

Researchers interested in using the data and analysis for scientific purposes can contact the corresponding author, Prof. Narges Khanjani.

## Abbreviations

CCHF: Crimean Congo Hemorrhagic Fever
SARIMA: Seasonal Autoregressive Integrated Moving Average
GAM: Generalized Additive Model
GAMM: Generalized Additive Mixed Model
ACF: Auto Correlation Function
PACF: Partial Auto Correlation Function
AR: Auto Regressive
AIC: Akaike Information Criterion
BIC: Bayesian Information Criterion
CCF: Cross-Correlation Function
VIF: Variance Inflation Factor
